# The phytochemical epigallocatechin gallate prolongs the lifespan by improving lipid metabolism, reducing inflammation and oxidative stress in high‐fat diet‐fed obese rats

**DOI:** 10.1111/acel.13199

**Published:** 2020-07-30

**Authors:** Hang Yuan, Yuqiao Li, Fan Ling, Yue Guan, Dandan Zhang, Qiushuang Zhu, Jinxiao Liu, Yuqing Wu, Yucun Niu

**Affiliations:** ^1^ Department of Nutrition and Food Hygiene College of Public Health Harbin Medical University Harbin Heilongjiang China; ^2^ Heilongjiang Health Development Research Center Heilongjiang China

**Keywords:** EGCG, free fatty acid, high‐fat dietary, lifespan, proteomics, transcriptome

## Abstract

We have recently reported that epigallocatechin gallate (EGCG) could extend lifespan in healthy rats. This study aimed to investigate the effects and mechanisms of a high dose of EGCG in extending the lifespan of obese rats. Ninety adult male Wistar rats were randomly divided into the control (NC), high‐fat (HF) and EGCG groups. Serum glucose and lipids, inflammation and oxidative stress were dynamically determined from adulthood to death, and the transcriptome and proteome of the liver were also examined. The median lifespans of the NC, HF and EGCG groups were 693, 599 and 683 days, respectively, and EGCG delayed death by 84 days in obese rats. EGCG improved serum glucose and lipids and reduced inflammation and oxidative stress associated with aging in obese rats induced by a high‐fat diet. EGCG also significantly decreased the levels of total free fatty acids (FFAs), SFAs and the n‐6/n‐3 ratio but significantly increased the n‐3 FFAs related to longevity. The joint study of the transcriptome and proteome in liver found that EGCG exerted its effects mainly by regulating the suppression of hydrogen peroxide and oxygen species metabolism, suppression of oxidative stress, activation of fatty acid transport and oxidation and cholesterol metabolism. EGCG significantly increased the protein expression of FOXO1, Sirt1, CAT, FABP1, GSTA2, ACSL1 and CPT2 but significantly decreased NF‐κB, ACC1 and FAS protein levels in the livers of rats. All the results indicate that EGCG extends lifespan by improving FFA metabolism and reducing the levels of inflammatory and oxidative stress in obese rats.

## Graphical abstract

This study investigates the effects and mechanisms of a high dose of epigallocatechin gallate (EGCG) in extending the lifespan of obese rats. We find that EGCG improved serum glucose, lipids, inflammation, oxidative stress associated with ageing in obese rats induced by a high‐fat diet, total free fatty acids (FFAs), transcriptome, and proteome. The results indicate that EGCG extends lifespan by improving FFA metabolism and reducing the levels of inflammatory and oxidative stress in obese rats.

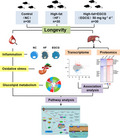

## 1 INTRODUCTION

Life expectancy is influenced by many factors. Among them, food is the foundation of lifespan. It is closely related to the morbidity, mortality and various health events of different diseases, and it is also an important factor affecting longevity both in animal and in human experiments (Piper, Skorupa, & Partridge, 2005; Wynder & Andres, 1994). However, a high‐fat diet can lead to obesity directly by aggravating the degree of chronic inflammation and oxidative stress, which can further reduce the lifespan of mice (Zhang et al., 2015).

Obesity is an escalating epidemic and may be attributed to the overconsumption of food and the imbalance of fatty acids. Several mechanisms have been suggested to explain the enhanced oxidative stress observed in subjects with obesity, including altered lipid and glucose metabolism, chronic inflammation, tissue dysfunction, hyperleptinaemia and abnormal postprandial ROS generation (Savini, Catani, Evangelista, Gasperi, & Avigliano, 2013). Obesity, including damage at the cellular and molecular levels, triggers an oxidative stress process, reduces quality of life and shortens human life expectancy (Stewart, Cutler, & Rosen, 2009). Previous studies have demonstrated that moderate obesity reduces life expectancy by 2–3 years and morbid obesity by an additional 8–10 years in prospective studies (Whitlock et al., 2009). Consequently, finding natural foods and phytochemicals that can ameliorate inflammation and oxidative stress caused by obesity has become an urgent issue.

Numerous clinical trials and animal studies have demonstrated that phytochemicals have many health benefits, including anti‐inflammatory and antioxidative stress; lowering serum glucose, serum lipids and blood pressure; improving immunity; and even extending lifespan (Holst & Williamson, 2008; Krzyzanowska, Czubacka, & Oleszek, 2010; Manach, Hubert, Llorach, & Scalbert, 2009; Scalbert et al., 2011). Epigallocatechin gallate (EGCG), the main and most abundant catechin in green tea, is a common phytochemical that is claimed to have many biologically active components. It has shown positive results for its anticarcinogenic, antioxidant, radical scavenging, anti‐inflammatory and lowering of blood glucose and lipid properties (Yang, Wang, Lu, & Picinich, 2009). It has also been shown to effectively reduce body fat in both animal experiments and human studies (Bose et al., 2008). The antiobesity properties of EGCG were explained by a decrease in food digestibility affecting substrate metabolism in the liver and intestinal mucosa, leading to increased postprandial fat oxidation and reduced incorporation of dietary lipids into tissues in mice (Friedrich, Petzke, Raederstorff, Wolfram, & Klaus, 2011). However, most of the studies related to dietary catechins remain in population epidemiology or short‐term intervention studies of low‐grade organisms, while studies of long‐term intervention using EGCG are rarely reported.

Early work has found that EGCG can maintain the health of rats through anti‐inflammatory and antioxidant activities and extend the lifespan of rats and has determined an effective dose of EGCG (25 mg kg^−1^ day^−1^） (Niu et al., 2013). However, it is not clear whether EGCG can combat damage to the body by an unhealthy high‐fat dietary pattern to a certain extent and play the role of prolonging the lifespan of the body. To this end, to determine the mechanism of extending lifespan in a high‐fat diet, this study aimed to investigate the effect of EGCG supplementation on the lifespan of rats fed a high‐fat diet; to dynamically monitor the level of inflammation, oxidative stress, serum lipids and glucose during the whole lifespan of healthy rats; to assess indicators that reflect liver and kidney function by pathological section; to detect the free fatty acids in serum; and finally to analyse the transcriptome sequencing and proteomics on the liver of aging rats.

## 2 RESULTS

### 2.1 EGCG prolongs the healthspan and lifespan of obese rats

**Figure 1:**
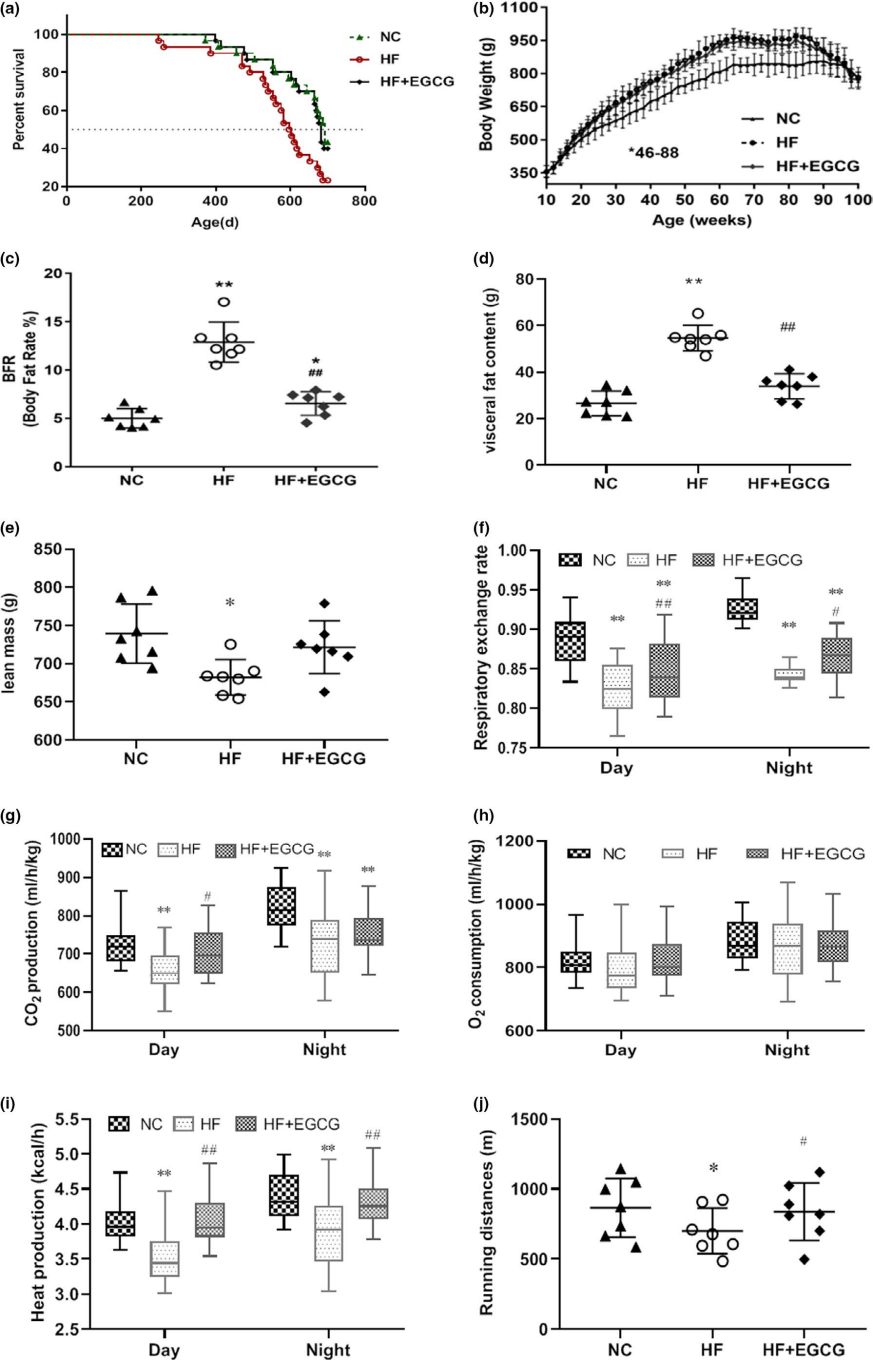
Effects of EGCG on lifespan, body weight, body fat rate, visceral fat content, lean mass and energy metabolism in obese rats. Ninety adult male Wistar rats were randomly divided into the normal control (n = 30), high‐fat diet (n = 30) and EGCG treatment groups (50 mg/kg/d, n = 30). The experiment lasted for 100 weeks. (a) Kaplan–Meier (log‐rank) survival curves (*p* = 0.043). (b) Body weight. (c) Body fat rate (n = 7). (d) Visceral fat content (n = 7). (e) Lean mass (n = 7). (f) Respiratory exchange rate (n = 7). (g) Carbon dioxide production (n = 7). (h) Oxygen consumption (n = 7). (i) Heat production (n = 7). (j) Running distances (n = 7). Data are presented as the means ± SD, ^*^ *p* < 0.05 or **p < 0.01 compared with NC; ^##^ *p* < 0.01 compared with HF

We compared the differences in lifespan between the three groups and found that the median lifespans were 693, 599 and 683 days for the NC, HF and HF+EGCG groups, respectively. The median lifespan of the HF group was significantly decreased by 14.00% compared to that of the NC group (*p* = 0.0238). The EGCG group significantly increased the lifespan by 12.74% compared to that of the HF group (*p* = 0.0313). There were also statistically significant differences between the three groups in the median lifespan (*p* = 0.043; Figure 1a).

We also measured body weight and composition, energy metabolism, systemic tumour burden, and liver and kidney damage in this study, which could be associated with aging (Tsubota, 2007). Compared to the NC group, the body weight of the HF group was significantly increased (from 16 weeks to 90 weeks), and EGCG significantly decreased the body weight from 46 weeks to 88 weeks compared to that of the HF group (Figure 1b). Body composition analysis at 100 weeks showed that the body fat ratio (BFR) and fat mass were significantly higher in the HF group than in the NC group, but lower in the EGCG group than in the HF group (Figure 1c,d). No change was observed in lean mass by EGCG (Figure 1e). Energy metabolism was measured in rats aged 64 weeks under the same average calorie intake. The respiratory exchange ratio and carbon dioxide production were significantly reduced, but heat production was significantly increased both during the day and night in the EGCG group compared with the HF group (Figure 1f,g,i), suggesting an increase in fat utilization induced by EGCG. Oxygen consumption was not different between the EGCG and HF groups (Figure 1h). In addition, physical performance tests were implemented and found that the running distances were significantly lengthened by EGCG in the treadmill test compared with distances of the HF group (Figure 1j), suggesting an improvement in the general fitness of rats.

**Figure 2:**
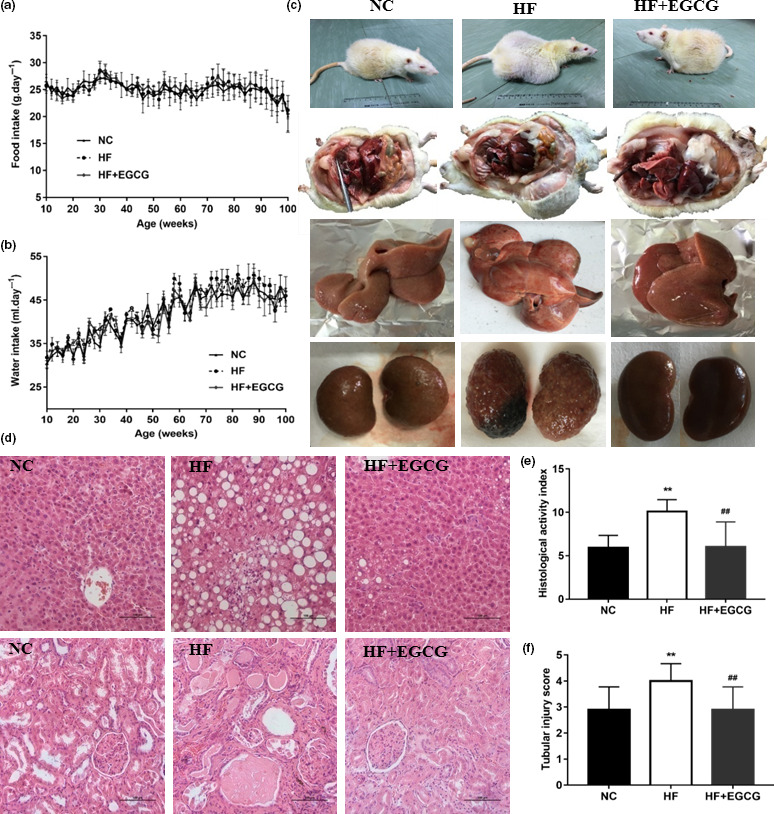
Epigallocatechin gallate prolongs the healthspan and lifespan of obese rats. (a) Food intake. (b) Water intake. (c) The variation in body type and visceral organ damage (100 weeks, n = 7). (d‐f) Pathological sections of liver and kidney, the hepatic HAI score and the renal tubular injury scores (100 weeks, n = 7). **p < 0.01 compared with NC; ^##^ *p* < 0.01 compared with HF

There were no significant differences in food intake and water intake between the three groups during the course of the experiment (Figure 2a,b). The major pathologies identified at necropsy suggested that rats in the EGCG group had a reduction in tumour occurrence, particularly subcutaneous sarcoma, compared with the HF group (Table S1). The appearance of animals and tissues at 100 weeks obviously showed variations in body type and visceral organ damage (Figure 2c). The pathological analysis at 100 weeks found that EGCG led to a reduction in the type and number of some pathologies, including inflammatory cell infiltration aspects of fibrosis, transparent degeneration and necrosis. Moreover, compared with the NC group, the renal tubules showed glomerular structure loss, necrosis, epithelial cell exfoliation and numerous protein tubules in the HF group, which were improved by the EGCG group (Figure 2d, Table S2). However, EGCG significantly reduced the tubular injury score of kidney pathology and the HAI score of the liver (Figure 2e,f). Altogether, these results suggest that EGCG could extend the healthspan and lifespan of adult obese rats by improving the levels of frailty, including body composition, systemic tumours, energy metabolism and liver and kidney damage.

### 2.2 EGCG reduced serum glucose, serum lipids, inflammation and oxidative stress and improved the function of the liver and kidney

**Figure 3:**
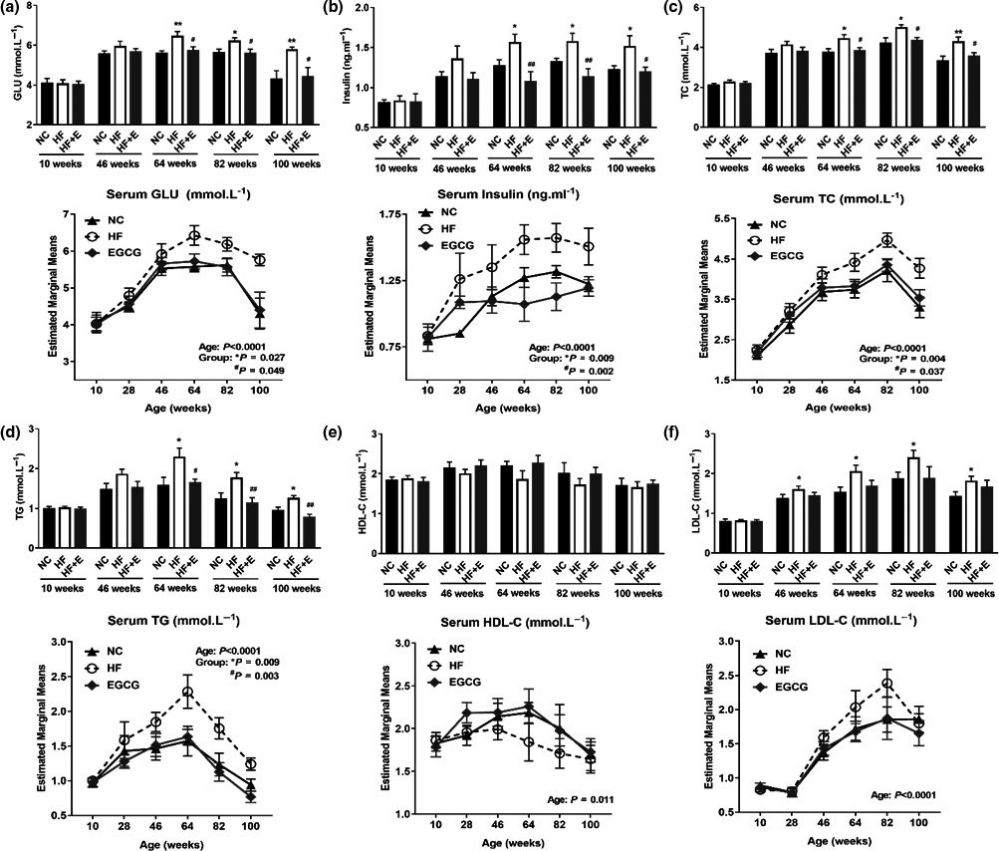
Epigallocatechin gallate reduced serum glucose, serum lipids, inflammation and oxidative stress and improved the function of the liver and kidney. (a) Serum glucose (GLU). (b) Serum insulin. (c) Serum total cholesterol (TC). (d) Serum triglycerides (TG). (e) Serum high‐density lipoprotein cholesterol (HDL‐C). (f) Serum low‐density lipoprotein cholesterol (LDL‐C). All values are the means ± SD, n = 7 for 100 weeks and n = 10 for other weeks.^*^ *p* < 0.05 or **p < 0.01 compared with NC; ^#^ *p* < 0.05 or ^##^ *p* < 0.01 compared with HF

Serum biomarkers were measured six times at 10, 28, 46, 64, 82 and 100 weeks of age during the whole life of rats. As age increased, serum glucose (GLU), insulin, total cholesterol (TC), triglycerides (TG) and low‐density lipoprotein cholesterol (LDL‐C) generally increased up to 82 weeks old, followed by a slight decline. The levels of GLU, insulin, TC, TG and LDL‐C were significantly increased in the HF group compared with the NC group. Additionally, compared with the HF group, EGCG obviously decreased the levels of serum GLU, insulin, TC and TG from 64 weeks and LDL‐C from 46 weeks. High‐density lipoprotein cholesterol (HDL‐C) showed no difference in the whole study among the three groups (Figure 3a–f).

The indicators including alanine aminotransferase (ALT), aspartate aminotransferase (AST), interleukin‐6 (IL‐6), tumour necrosis factor‐α (TNF‐α), superoxide dismutase (SOD), reactive oxygen species (ROS) and malondialdehyde (MDA) showed an overall upward trend, but glutathione peroxidase (GSH) had a downward trend with the gradual increase in age. Compared with the NC group, serum ALT (from 64 weeks) and AST (from 82 weeks) were significantly increased in the HF group (*P < *0.05). In the EGCG group, ALT and AST levels were significantly decreased from 82 weeks compared with the HF group (*P < *0.05). The levels of IL‐6, TNF‐α and ROS associated with inflammation and oxidative stress were significantly increased from 82 weeks in the HF group compared with the NC group (*P < *0.05). Compared with the HF group, IL‐6 (from 82 weeks), TNF‐α (from 64 weeks) and ROS (from 82 weeks) were significantly decreased in the EGCG group (*P < *0.05). From 28 weeks old to the end of the experiment, SOD levels in the HF and EGCG groups were significantly lower than those in the NC group, and the SOD level in the EGCG group was significantly higher than that in the HF group (*P < *0.05). Although MDA and GSH showed a slight trend of improvement, there was no significant difference in the EGCG intervention group (Table 1). These results indicated that EGCG extended the median lifespan possibly by improving glycolipid metabolism, alleviating liver and kidney function and reducing inflammation and oxidative stress associated with the high‐fat diet.

**Table 1:** Effects of EGCG treatment on various serum biochemistry characteristics

Variable	Group	Age(weeks)						*P* value (age)
		10 (n = 10)	28 (n = 10)	46 (n = 10)	64 (n = 10)	82 (n = 10)	100 (n = 7)	
ALT (U/L)	NC	29.43 ± 4.50	29.57 ± 3.15	33.75 ± 8.83	33.50 ± 4.24	34.20 ± 2.20	19.63 ± 5.24	<0.0001
	HF	28.62 ± 4.80	33.57 ± 3.74	38.29 ± 9.78	41.56 ± 9.36**	51.25 ± 9.88**	47.50 ± 9.92**	
	EGCG	30.78 ± 3.22	31.43 ± 5.65	36.63 ± 9.61	36.86 ± 6.96	37.67 ± 9.67^#^	25.50 ± 9.89^#^	
AST (U/L)	NC	138.50 ± 18.24	144.88 ± 20.02	145.50 ± 35.13	153.75 ± 27.47	164.38 ± 22.53	160.75 ± 18.14	<0.0001
	HF	127.46 ± 16.77	156.38 ± 23.53	162.00 ± 27.01	178.83 ± 12.25	190.00 ± 30.08*	186.25 ± 19.01*	
	EGCG	147.84 ± 18.18	151.63 ± 19.99	152.00 ± 28.73	168.40 ± 41.16	160.38 ± 17.7*	135.50 ± 21.64^*##^	
IL−6 (pg mL^−1^)	NC	9.22 ± 1.21	9.00 ± 1.43	9.51 ± 1.99	10.97 ± 4.92	12.42 ± 3.12	9.89 ± 2.78	<0.0001
	HF	9.15 ± 1.06	12.90 ± 4.10	14.39 ± 4.13	11.38 ± 1.44	16.24 ± 2.86*	13.83 ± 2.66**	
	EGCG	9.26 ± 0.34	11.71 ± 0.21	11.16 ± 0.95	12.46 ± 0.55	13.60 ± 1.54^#^	11.75 ± 1.35^#^	
SOD (units)	NC	1.48 ± 0.11	1.25 ± 0.18	1.13 ± 0.19	0.96 ± 0.26	0.99 ± 0.24	0.99 ± 0.05	<0.0001
	HF	1.51 ± 0.08	0.92 ± 0.21**	0.91 ± 0.16*	0.74 ± 0.15*	0.73 ± 0.27*	0.84 ± 0.20*	
	EGCG	1.44 ± 0.13	1.86 ± 0.16^**##^	1.66 ± 0.26^**##^	1.42 ± 0.24^**##^	1.43 ± 0.23^*##^	1.29 ± 0.03^**##^	
TNF‐α (pg mL^−1^)	NC	20.56 ± 0.31	20.92 ± 0.51	21.69 ± 3.73	23.48 ± 4.73	24.95 ± 2.76	23.33 ± 2.14	<0.0001
	HF	21.08 ± 0.82	21.35 ± 2.59	23.77 ± 3.81	23.82 ± 5.43	27.32 ± 1.51*	25.32 ± 2.65*	
	EGCG	21.36 ± 0.42	21.22 ± 1.89	22.4 ± 2.84	19.34 ± 1.08^*#^	18.54 ± 0.58^**##^	18.31 ± 1.14^**##^	
ROS (U mL^−1^)	NC	37.21 ± 2.42	38.66 ± 1.40	41.01 ± 5.03	43.32 ± 5.45	43.96 ± 1.12	39.78 ± 3.53	0.047
	HF	36.55 ± 3.03	41.32 ± 4.59	44.33 ± 7.16	44.79 ± 1.46	45.55 ± 2.92*	43.36 ± 3.11*	
	EGCG	37.43 ± 1.19	40.85 ± 6.85	43.36 ± 5.92	36.13 ± 5.64	35.03 ± 6.33^**##^	36.87 ± 2.18^*#^	
MDA (μmol L^−1^)	NC	1.45 ± 0.20	1.67 ± 0.38	1.66 ± 0.26	1.45 ± 0.42	1.95 ± 1.15	1.42 ± 0.13	0.006
	HF	1.39 ± 0.38	1.70 ± 0.98	1.99 ± 0.75	1.64 ± 0.38	1.99 ± 0.30	1.62 ± 0.30	
	EGCG	1.42 ± 0.24	1.02 ± 0.60	1.72 ± 0.34	1.59 ± 0.87	1.82 ± 0.18	1.39 ± 0.49	
GSH (μmol L^−1^)	NC	8.43 ± 1.07	8.17 ± 2.49	6.37 ± 1.63	7.92 ± 1.53	6.96 ± 3.31	4.72 ± 1.49	<0.0001
	HF	7.79 ± 1.24	7.75 ± 1.18	5.99 ± 2.13	6.43 ± 1.05	5.92 ± 1.48	4.69 ± 2.78	
	EGCG	8.17 ± 1.16	6.73 ± 1.71	7.40 ± 2.11	8.19 ± 2.72	7.46 ± 2.92	5.65 ± 2.27	

Abbreviations: ALT, alanine aminotransferase; AST, aspartate aminotransferase; GSH, glutathione; IL‐6, interleukin‐6; MDA, malondialdehyde; ROS, reactive oxygen species; SOD, superoxide dismutase; TNF‐α, tumour necrosis factor‐a.

All values are mean ± SD.

*p* value in the last column is the statistical result of two‐factor repeated‐measures ANOVA over time.

^#^ *p* < 0.05,

^##^ *p* < 0.01 vs HF group.

* *p* < 0.05,

** *p* < 0.01 vs NC group.

### 2.3 EGCG decreases serum‐free fatty acid (FFA) levels but increases N‐3 FFA levels

To further characterize the metabolic changes in the three groups, we analysed the fatty acid profiles in the chow diet and serum at 46 and 100 weeks of age. The composition of saturated fatty acid (SFA) and monounsaturated fatty acid (MUFA) levels was significantly higher, and polyunsaturated fatty acids (PUFAs) were significantly lower in normal chow than in high‐fat chow (Figure S1A). All the levels of serum FFAs except n‐3 FFAs were significantly increased in the HF group compared with the NC group, and EGCG significantly decreased these FFA levels at both 46 and 100 weeks of age (Figure S1B and Table S3). In addition, almost all kinds of total serum‐free fatty acids in the EGCG group were significantly decreased compared with those in the HF group. Of interest, only the proportion of serum N‐3 FFAs, especially α‐linolenic acid, in the EGCG group was obviously increased compared with the HF group, which was lower than that in the normal group with increasing age (Table S3). Taken together, these results suggested that EGCG improved the physiological and metabolic FFA metabolism associated with longevity and especially increased serum n‐3 levels in obese rats.

### 2.4 Impacts of lifelong EGCG treatment on the liver transcriptome and proteome

**Figure 4:**
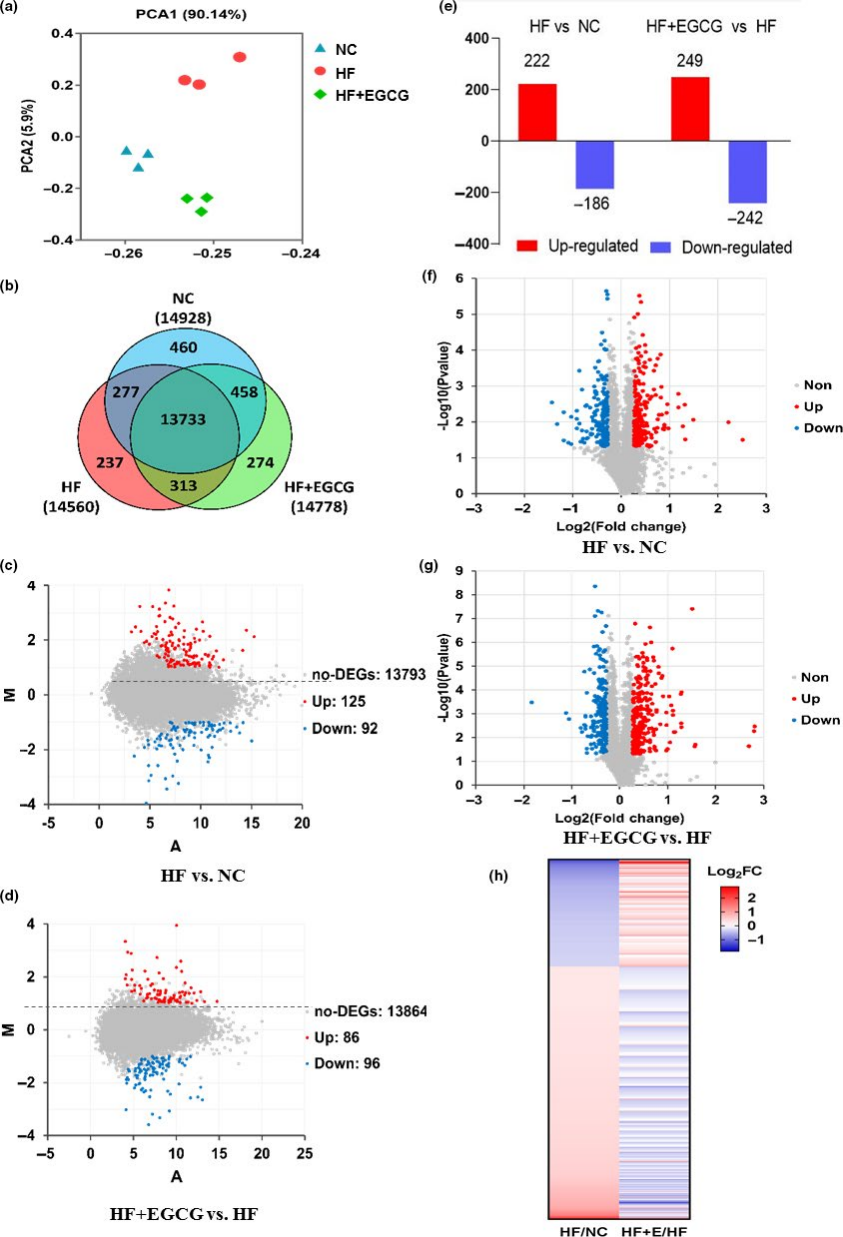
Effects of EGCG on the liver transcriptome and proteome using mRNA sequencing and iTraq proteomic methods. (a) PCA for gene quantification data derived from RNA sequencing. (b) Venn diagram analysis for co‐expressed genes. (c) MA plot analysis for differentially expressed genes; we marked the points with *p* < 0.05 and log_2_ FC of transcripts >1 or <−1 in the HF group vs NC. (d) MA plot analysis for differentially expressed genes in the EGCG group vs HF. (e) The protein expression changes based on a *P* < 0.05. (f) Volcano plot analysis for differentially expressed proteins depicting the points with *p* < 0.05, and the greater the fold change is, the farther away from the vertical centreline in the HF group vs NC. (g) Volcano plot analysis for differentially expressed proteins in the EGCG group vs HF. (h) Heap map for classification of the differentially expressed proteins. n = 3 for each group and aged 100 weeks, and all red dots represent upregulated, blue dots represent downregulated, and grey dots represent no obvious changes in the figures

To clarify how the EGCG group extended the lifespan of the obese rats, mRNA sequencing and iTraq proteomics were performed on liver samples of aging rats at 100 weeks. PCA found that the three groups were significantly separated, which indicated that their transcriptomes were dramatically different (Figure 4a). A further Venn diagram analysis revealed 13,733 transcripts as co‐expressed transcripts in the three groups (Figure 4b). We identified the differentially expressed genes according to the parameters of a significant difference (*p* < 0.05) and the transformed fold change (log_2_ FC) of transcripts >1 or < −1 between the two groups. A total of 125 genes were upregulated, and 92 genes were downregulated in the HF group versus the NC group, and 86 genes were upregulated and 96 genes were downregulated in the EGCG group versus the HF group (Figure 4c,d, red represents upregulated, blue represents downregulated, and grey represents non‐regulated, the greater the fold change is, the farther away from the horizontal centreline). Gene Ontology (GO) enrichment analyses for significantly altered genes in the EGCG group identified that the gene pathways were markedly downregulated, such as suppression of hydrogen peroxide and oxygen species metabolism, suppression of lipid metabolism and suppression of oxidative stress (Table 2, Figure S2).

**Table 2:** Pathway and GO regulated by EGCG versus HF in transcriptome and proteome

Transcriptome			Proteome		
Pathway	*P* Value	Fold Enrichment/Log_2_ (FE)	Pathway	*P* Value	Fold Enrichment
Hydrogen peroxide metabolic process^a^	＜0.0001	−20.436	PPAR signalling pathway^b^	＜0.0001	7.073
Reactive oxygen species metabolic process^a^	＜0.0001	−21.451	Fatty acid degradation^b^	＜0.0001	4.030
Lipid metabolism^a^	0.0025	−3.271	positive regulation of lipid catabolic process	＜0.0001	2.330
Suppression of oxidative stress^a^	0.0119	−14.867	lipid metabolic process^a^	0.0001	24.712
			fatty acid oxidation^a^	0.0041	1.296
			positive regulation of lipid metabolic process^a^	0.0077	6.877
			(long‐chain) fatty acid transport^a^	0.0101	2.318
			cholesterol metabolic process^a^	0.0046	7.697

^a^ Gene ontology.

^b^ KEGG pathway.

Likewise, a global proteomic analysis found that the total number of differentially expressed proteins in the EGCG group compared with the HF group was 491, with 249 upregulated and 242 downregulated (Figure 4E), and the others detailed differentially expressed proteins between two groups are shown in Figure S3A. Volcano plot analysis for the differentially expressed proteins was performed between the two groups. Red represents upregulated, blue represents downregulated, and grey represents non‐regulated based on a *p *< 0.05, and the greater the fold change is, the farther away from the vertical centreline (Figure 4f,g and Figure S3b). Visually, compared with the NC group, the upregulated proteins in the HF group were downregulated by EGCG treatment. However, the downregulated proteins in the HF group were upregulated by EGCG (Figure 4h). In addition, nearly half of the proteins were downregulated in both the EGCG and HF groups, but surprisingly, most proteins were uniquely upregulated in the EGCG group (Figure S3c). Kyoto Encyclopedia of Genes and Genomes (KEGG) enrichment analyses identified that PPAR signalling and fatty acid degradation were upregulated by comparing the EGCG group with the HF group. GO analyses identified that the protein pathways were markedly upregulated, including lipid metabolism and catabolism, fatty acid oxidation, long‐chain fatty acid transport and cholesterol metabolism (Table 2, Figure S3d,e).

**Figure 5:**
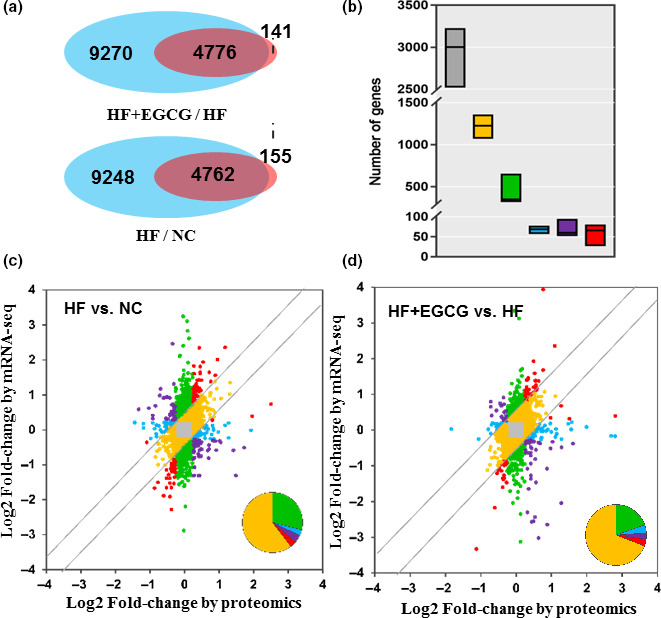
Correlation analysis of the transcriptome and proteome. (a) Counts of co‐expressed genes and proteins. The blue represents only genes, the pink represents only the proteome, and the overlap represents the co‐expressed genes and proteins. (b) Analysis of co‐expressed genes and proteins. Different colours represent different meanings, such as grey (fold change ≤1.5 for both mRNA and protein), yellow (fold change ≥1.5 for both mRNA and protein), green (fold change ≥1.5 for mRNA but ≤1.5 for protein) and blue (fold change ≤1.5 for mRNA but ≥1.5 for protein). Red genes represents those with a consistent direction but variable magnitude of change (≥1.5‐fold) between the regions at the protein and RNA level, while purple genes disagree in the direction of change between the mRNA and protein. (c) Different genes or proteins are coloured based on their fold changes of mRNA and protein in the HF group vs NC. (d) Different genes or proteins are coloured based on their fold changes of mRNA and protein in the EGCG group vs HF. n = 3 for each group and aged 100 weeks

To determine the key role of gene and protein pathways induced by EGCG treatment, we integrated gene‐level mRNA expression data with quantitative proteomics. The results of the peptide mapping indicated that 4762 genes and proteins were co‐expressed between the HF group and the NC group, and 4776 genes and proteins were co‐expressed between the EGCG group and the HF group (Figure 5a). The distribution of differential genes and proteins is shown in Figure 5b. For further analyses of co‐expressed genes and proteins, 25.5% showed larger (fold change ≥1.5) region‐specific expression differences in the same direction, and they were considered ‘agree’ and ‘partially agree’ (same direction of change, a different magnitude of fold change). In addition, 61.2% of co‐expressed genes and proteins were found with fold changes ≤1.5 for mRNA and protein, 9.2% with fold change ≥1.5 for mRNA but ≤1.5 for proteins, and 1.4% with fold change ≤1.5 for mRNA but ≥1.5 for proteins in the correlation analysis. A small fraction (1.4%) of genes disagreed in the direction of change between mRNA and protein (Figure 5c,d). All results suggested that there was good consistency in the transcriptome and proteomics changes in rat liver induced by EGCG, especially for the regulation of lipid metabolism.

### 2.5 EGCG improved the proteins related to lipid metabolism, inflammation and oxidative stress

**Figure 6:**
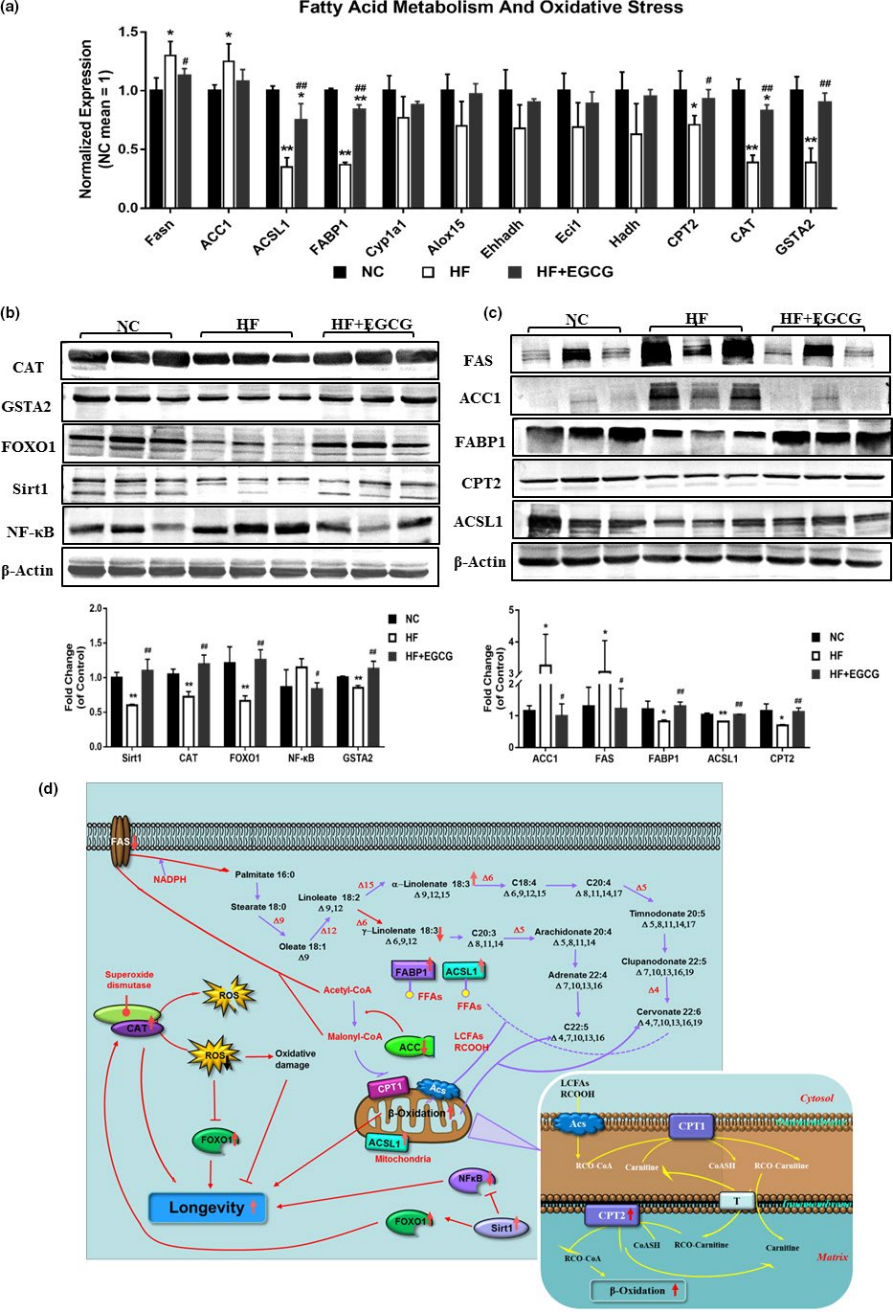
Further verification of the changes in the transcriptome and proteome. (a) The mRNA levels of Fasn, ACSL1, FABP1 CPT2, CAT and GSTA2 were measured in the liver by quantitative real‐time RT‐PCR. (b) The protein expression levels of CAT, GSTA2, FOXO1, SIRT1, NF‐κB, FAS, ACC1, FABP1, CPT2 and ACSL1 were measured in the liver by Western blot methods. A representative loading control is shown for each case (n = 3). (c) Molecular pathway analysis of EGCG extends the lifespan in obese rats. Data were presented as the ratios of target protein to β‐actin. All values are the means ±SD. ^*^ *p* < 0.05 or **p < 0.01 compared with NC; ^#^ *p* < 0.05 or ^##^ *p* < 0.01 compared with HF

To determine whether these transcriptional and protein patterns were consistent with reality and not by accident, the genes of fatty acid synthase (Fasn), acetyl‐CoA carboxylase (ACC1), long‐chain acyl‐CoA synthetase 1 (ACSL1), fatty acid binding protein‐1 (FABP1), cytochrome P450 la1 (Cypla1), arachidonate 15‐lipoxygenase (Alox15), enoyl‐coenzyme A, hydratase/3‐hydroxyacyl coenzyme A dehydrogenase (Ehhadh), enoyl‐CoA delta isomerase 1 (Eci1), hydroxyacyl‐CoA dehydrogenase (Hadh), carnitine palmitoyltransferase II (CPT2), catalase (CAT) and glutathione s transferase alpha 2 (GSTA2) that are related to lipid metabolism and longevity and were previously found to be functionally enriched were validated. The results showed that EGCG significantly decreased the mRNA level of Fasn and increased the mRNA levels of ACSL1, FABP1, CPT2, CAT and GSTA2, which were roughly the same as those of the transcriptome (Figure 6a). In addition, some proteins that were classically recognized to be associated with lipid metabolism, inflammation, oxidative stress and longevity were also validated. The protein levels of CAT, GSTA2, forkhead box O1 (FOXO1), silent information regulator 1 (SIRT 1), FABP1, CPT2 and ACSL1 were significantly increased by EGCG compared with the HF group, along with the protein levels of free fatty acid synthase (FAS), acetyl‐CoA carboxylase (ACC1) and nuclear factor kappa‐B (NF‐κB) (Figure 6b).

Integrating all the data, we can obtain a few clues illustrating that EGCG extended lifespan in obese rats. Admittedly, our research confirmed that EGCG extends the lifespan by increasing the expression of the FOXO1 protein and decreasing ROS generation by increasing CAT protein expression and indirectly increasing CAT protein expression by upregulating Sirt1 and FOXO1 protein expression. In addition, EGCG extended the lifespan by attenuating inflammation through inhibiting NF‐κB activation. Finally, EGCG extended the lifespan by increasing FABP1, ACSL1, CPT2 and ACSL1 protein expression for fatty acid transport and oxidation and decreasing ACC1 and FAS protein expression for fatty acid synthesis (Figure 6c). Altogether, these results indicate that EGCG could extend lifespan in obese rats by improving inflammation, oxidative stress and lipid metabolism, especially free fatty acid metabolism, in obese rats.

## 3 DISCUSSION

The objective of this study was to determine whether EGCG treatment in a high‐fat diet setting increased the longevity and median lifespan of rats. The reason for studying a high‐fat diet is that the impact of a high‐fat diet on longevity is even more serious (Seidell, 1998). The results clearly demonstrated that lifespan was increased in rats fed an EGCG treatment compared with a high‐fat diet.

Some studies have found that EGCG is not toxic at doses below 500 mg/kg in rats or dogs, and it is safe for healthy individuals to consume 752 mg/kg or 800 mg/day EGCG with no severe symptoms or adverse effects (Isbrucker, Edwards, Wolz, Davidovich, & Bausch, 2006; Fernandes et al., 2018; Sherry Chow et al., 2003). In addition, the oral bioavailability of EGCG is estimated to be approximately 0.1 to 0.3% in rats and humans (Kuriyama et al., 2006; Noguchi‐Shinohara et al., 2014). Therefore, to better improve the hazard of a high‐fat diet on health, chronic disease and lifespan, we increased the intervention dose of EGCG to 50 mg kg^−1^ d^−1^ from 25 mg kg^−1^ d^−1^ that was used in our previous study. This dose is approximately 500 mg kg^−1^ d^−1^ when it is extrapolated to humans using the method of specific surface area according to the average human weighing 60 kg. A study found that by consuming an average of three 8 oz. cups of green tea daily, the estimated daily intake of EGCG can reach approximately 560 mg/d (Jiang, Webster, Cao, & Shao, 2018). Of course, it could also be developed into medicine, health foods or nutrition supplements as a concentrate to increase the dosage of EGCG. To make this process closer to people's habit of drinking tea, we administered EGCG to the rats by drinking water every night when they were active. To prevent possible differences, the process of changing the water was synchronized for all the groups. The result was that there was no significant difference in drinking, which showed that EGCG, which is slightly bitter, did not affect the amount of water intake in rats. To exclude any potential effects of EGCG in chow, all rats were fed a synthetic diet according to the AIN‐93 formula without EGCG. As the median lifespan completely reflects the whole lifespan, which has been widely applied as a standard index for longevity investigation in many studies (Aihie Sayer, Dunn, Langley‐Evans, & Cooper, 2001; Bernardes de Jesus et al., 2012), we ended the experiment when over half of the rats died in each group.

The median lifespan in the HF group was significantly decreased to 599 days compared with 693 days in the NC group and was significantly increased to 683 days in the EGCG group compared with the HF group. Some studies have certified that EGCG increases the lifespan of *Caenorhabditis elegans* or animal models induced by external factors. Furthermore, some epidemiologic studies have shown that tea consumption can prolong life by avoiding cardiovascular disease and cancer (Arab, Khan, & Lam, 2013; Khan & Mukhtar, 2018). However, Strong et al. (2013) reported that green tea extract might diminish the risk of midlife deaths in females and has no effect on locomotor activity as a measure of healthspan and the whole lifespan of old male and female genetically heterogeneous mice. The reason for the discrepancy could be that the exact EGCG concentration in the green tea extract is unknown. Obesity and overweight could increase all‐cause mortality and reduce lifespan, which was related to the body's total fat mass. Our study also confirmed that a high‐fat diet increased visceral fat mass in rats, and the EGCG group had significantly less fat mass than the HF group. Furthermore, we also found that EGCG improved the function of multiple organs in autopsy and pathological experiments, especially the inhibition of tumours such as nasopharyngeal carcinoma.

Lifespan is associated with age‐related chronic diseases such as hyperlipidaemia, diabetes and cardiovascular disease (Rajpathak et al., 2011). Blood glucose and lipids are some of the most critical factors affecting the above‐mentioned chronic diseases. In fact, the overall trends of serum glucose and serum lipids except for HDL‐C from adulthood to old age in rats increased with increasing age, but there was a slight decrease beginning at the age of 82 weeks during the whole experiment course. The possible reason was due to the possibility of invisible diseases and organ damage, including weakness and dysphagia, which cause weight loss by reducing the animal's intake. Coincidentally, a similar trend was found in other studies. Our results also showed that EGCG could decrease the levels of serum GLU, insulin, TC, TG and LDL in obese rats, which was consistent with many other studies (Ashida et al., 2004; Park et al., 2011; Sherry Chow et al., 2003). In addition, many studies have confirmed that EGCG can improve insulin sensitivity and impaired glucose tolerance as measured by the insulin tolerance test and oral glucose tolerance test in both animal studies and human experiments (Ren, Yang, Yi, Zhang, & Yang, 2020; Torres, Cogliati, & Otton, 2019; Ueda‐Wakagi, Nagayasu, Yamashita, & Ashida, 2019; Venables, Hulston, Cox, & Jeukendrup, 2008). Therefore, we speculate that EGCG may increase the lifespan of obese rats by improving glucose and lipid metabolism.

Aging and longevity are associated with pro‐inflammatory cytokines and oxidative damage, and many studies have proven that it is an effective way to delay aging or prolong lifespan by improving inflammation and oxidative stress in animal and human experiments (Starr, Evers, & Saito, 2009). In our study, the major sources of oxidative stress and inflammatory molecules, including IL‐6, TNF‐alpha, SOD, GSH, ROS and MDA, showed an overall upward trend with the gradual increase in age in rats. EGCG significantly decreased serum IL‐6, TNF‐α and ROS levels in rats induced by the high‐fat diet. Therefore, it is suggested that EGCG extends lifespan in obese rats possibly by improving inflammation and oxidative stress.

Many population trials have proven that high levels of saturated fatty acids are risk factors for cardiovascular and chronic diseases, including hyperlipidaemia, diabetes mellitus and hypertension. The reason may be that SFAs increase inflammation and oxidative stress levels or increase serum cholesterol levels by increasing the activity of 3‐hydroxy‐3‐methylglutaryl coenzyme A reductase in the liver. EGCG significantly decreased the SFA levels in obese rats induced by a high‐fat diet. In addition, EGCG also significantly increased the levels of n‐3 fatty acids and decreased the ratio of n‐6/n‐3 fatty acids, which verified the function of inhibiting inflammation, reducing LDL and TG in serum and the risk of cardiovascular disease, delaying senescence and prolonging lifespan (Lai et al., 2018; Zhu, Ferrara, & Forman, 2018). In addition, EGCG increased the level of α‐linolenic acid and competitively inhibited γ‐linolenic acid, which also plays a key role in improving free fatty acid metabolism or extending the lifespan.

The liver is the main organ for body metabolism, and many studies have used the liver for omics studies to reflect systemic indicators (Karunadharma et al., 2015; Templeman et al., 2017), so we used the liver for transcriptome and proteome analyses. Protein interactions were analysed for genes that were highly expressed or played a key role. After analysing their functions, we illustrated how EGCG might prolong the lifespan in obese rats with high‐fat dietary patterns from several aspects. The joint study of transcriptomics and proteomics found that EGCG exerted its effects mainly by regulating suppression of hydrogen peroxide and oxygen species metabolism, suppression of oxidative stress, and activation of fatty acid transport and oxidation and cholesterol metabolism. The key mRNAs and proteins altered by EGCG were verified by RT‐PCR and Western blot experiments.

EGCG extended lifespan by directly increasing the protein expression of Sirt1 and FOXO1 related to longevity and possibly increased FOXO1 activity by reducing oxidative stress and ROS generation by increasing the mRNA and protein levels of CAT. Likewise, EGCG inhibited NF‐κB protein expression, which reduced inflammatory reactions (Campo et al., 2008; Srinivasan, Kriete, Sacan, & Jazwinski, 2010). Furthermore, the main idea we wanted to illustrate clearly was that EGCG might also play a role by altering fatty acid metabolism. We confirmed that EGCG inhibited the activity of FAS, reducing the catalysis of the synthesis of palmitate from acetyl‐CoA and malonyl‐CoA in the presence of NADPH. Furthermore, EGCG inhibited the activity of the ACC1 protein, which led to reduced malonyl‐CoA production and suppression of FFA synthesis and increased the protein expression of FABP1 and CPT2, which promoted the transport and oxidation of free fatty acids. Of course, many studies have fully proven that EGCG has anti‐inflammatory and antioxidation properties and improves lipid metabolism in animal experiments and human trials. In this study, we only found that the mechanism of prolonging the lifespan of rats fed EGCG for a long time may be closely related to the above effects. The molecular mechanisms of EGCG on extending lifespan and how to regulate lipid metabolism, inflammation and oxidative stress remain to be further verified.

## 4 EXPERIMENTAL PROCEDURES

### 4.1 Animals

Ninety adult male SPF Wistar rats, 8 weeks old, were obtained from Vital River Laboratory Animal Technology Company LTD. The animals were individually housed in stainless steel cages in a room at 21 ± 2°C, humidity 50 ± 5% on a 12‐h light/dark cycle with free access to food and water. After a one‐week acclimatization period, the animals were randomly divided into three groups: the normal control group (NC), high‐fat dietary group (HF) and high‐fat dietary EGCG treatment group (HF+EGCG, 50 mg kg^−1 ^d^−1^) (n = 30 in each group). EGCG (>95%, HPLC) was obtained from Medherb Biotechnology Co., Ltd. An amount of EGCG was added to the drinking water according to the weight of the rats, to simulate the method of human drinking tea. To ensure the dosage of EGCG was the same in each group, a little water containing EGCG before the addition of more water was fed to rats first. The rats were observed twice daily by the veterinary staff and animal care staff during their lifespan. Frequent communication regarding animal health issues occurred among all personnel involved in daily care, and the precise date of death of each rat was recorded. All experiments were approved by the Institutional Animal Care and Use Committee of Harbin Medical University and were conducted in compliance with the animal use guidelines (SCXK (Jing) 2016‐0006).

All rats received a regular AIN‐93 M diet (casein 14%, L‐cystine 0.18%, soybean oil 4%, corn starch 46.57%, corn dextrin 15.5%, sucrose 10%, cellulose 5%, choline bitartrate 0.25, vitamin mix AIN‐93 1% and mineral mix AIN‐93 3.5%) in this experiment. High‐fat feed (35% calories from fat) was formed from the basal AIN‐93 M diet by replacement of corn starch with 10% lard and 3.4% soybean oil. Food and water consumption and body weights were assessed by routine measurements once a week. Serum glucose and lipids, inflammation, oxidative stress, biochemistry variables and free fatty acid were determined at 10, 28, 46, 64, 82 and 100 weeks old. At the end of the experiment, the main fat weight of the surviving rats, such as perirenal fat, peritesticular fat and subcutaneous fat, was weighed and the BFR and visceral fat content were calculated. The lean mass was calculated as the sum of body weight minus perirenal fat, peritesticular fat and subcutaneous fat.

### 4.2 Liver and kidney pathology

Paraformaldehyde‐fixed paraffin sections of the liver and kidney were stained with haematoxylin and eosin (H&E) after washing with distilled water, dehydration, wax penetration, embedding, sectioning, spreading, dewaxing and rehydration, dyeing and sealing. Histological examinations were performed by pathologists blinded to the conditions. At least 5–10 fields were reviewed for each slide. The histological changes in kidney were scored by counting the percentage of tubules that displayed cell necrosis, loss of brush border, cast formation and tubule dilatation as follows: 0, none; 1, <10%; 2, 11%25%; 3, 26%–45%; 4, 46%–75%; and 5, >76% (Oh et al., 2008). The histological diagnosis of the liver was based on accepted criteria and the histological activity index (HAI) (Knodell et al., 1981). Briefly, HAI represents the sum of the scores attributed to the necroinflammatory lesions, that is, periportal and bridging necrosis (0–10), intralobular degeneration and focal necrosis (0–4), portal inflammation (0–4) and fibrosis (0–4).

### 4.3 Blood chemistry

Rats were deprived of food for 8 h prior to serum collection, which was used for the measurement of all blood chemistry. GLU, TC, TG, HDL‐C, LDL‐C, AST and ALT (Co‐Health Laboratories Co. Ltd) were measured using a ROCHE Modular P800 Automatic Biochemical Analyzer (Roche Diagnostics). SOD, GSH, MDA and ROS were measured with commercial kits using enzymatic methods (Beyotime Biotechnology). Insulin, TNF‐α and IL‐6 (R&D Systems Europe) were assayed using an enzyme‐linked immunosorbent assay (ELISA) with commercial kits, according to the manufacturers’ instructions.

### 4.4 Free fatty acid measurement

Serum FFA profile was measured using gas chromatography–mass spectrometry method (GC‐MS) described previously in article (Liu, Li, & Guan, 2010). Firstly, we purchased 16 fatty acid standards from Sigma (≥ 99% purity), including myristic acid (14:0), palmitic acid (16:0), palmitoleic acid (16:1n‐7), stearic acid (18:0), oleic acid (18:1n‐9), linoleic acid (18:2n‐6), linolenic acid (18:3n‐3), γ‐linolenic acid (γ‐18:3n‐6), cris‐6,9,12,15‐octadecatetraenoic acid (18:4n‐3), eicosadienoic acid (20:2n‐6), cris‐8,11,14 (20:3n‐6), arachidonic acid (20:4n‐6), cis‐5,8,11,14,17‐eicosapentaenoic acid (20:5n‐3), cris‐7,10,13,16‐adrenic acid (22:4n‐6), cis‐7,10,13,16,19‐docosapentaenoic acid (22:5n‐6) and cis‐4,7,10,13,16,19‐docosahexaenoic acid (22:6n‐3). Stock solutions of all the above fatty acids, internal standards (heptadecanoic acid) and calibration samples (spiking with 16 different concentrations of fatty acids standards) were prepared.

Briefly, aliquots (200 μl) of serum were spiked with an internal standard working solution (200 μl heptadecanoic acid C17:0 200 μg/ml), and 1 ml 0.05% H_2_SO_4_ was added to deposit protein. FFA was extracted using 3 ml ethyl acetate, mixed for 60 s (using a vortex mixer) and centrifuged at 4000 × g for 10 min at room temperature. The ethyl acetate phase was evaporated to dryness under N2. Following the addition of 2 ml and 10% H2SO4‐CH3OH, and incubation in 62°C water bath for 2 h, 2 ml saturated sodium chloride and 2 ml hexane were sequentially added and mixed for 60 s to obtain the fatty acid methyl esters. Samples were evaporated to dryness under N2 gas, and 100 μl hexane was added to each tube prior to analysis.

Free fatty acid concentrations of all standard and serum samples were determined using TRACE gas chromatograph with a Polaris Q mass spectrometer (Thermo Finnigan; GC–MS). A split injector (the split ratio being 1:10) at 230°C was used to add the sample (1.0 μl) onto a J&W DB‐WAX (30 m × 0.25 mm I.D., 0.25 μm film thickness) capillary column and fatty acid methyl esters were separated at constant flow article (Liu et al., 2010). The ion trap mass spectrometer was operated under electron bomb ionization (EI) mode. Mass spectra of m/z 30–450 were collected using full‐scan mode with 0.58 s/scan velocity. The solvent delay time was 5 min, source temperature was 230°C with the electron energy at 70 eV, limit of detection (LOD) was defined as lowest concentrations with signal‐to‐noise (S/N) ratios of 10, and all calibration samples showed high repeatability.

### 4.5 Transcriptome process and bioinformatics analysis

In our project, we sequenced 9 liver samples (n = 3 in each group) on Illumina HiSeq Platform in total. Agilent 2100 Bioanalyzer (Agilent RNA 6000 Nano Kit) was used to do the total RNA sample QC. mRNAs are isolated from total RNA with oligo (dT) method. Then, the mRNAs are fragmented under certain conditions. Then first‐strand cDNA and second‐strand cDNA are synthesized. cDNA fragments are purified and resolved with EB buffer for end reparation and single nucleotide A (adenine) addition. After that, the cDNA fragments are linked with adapters. These cDNA fragments with suitable sizes are selected for the PCR amplification. Agilent 2100 Bioanalyzer and ABI StepOnePlus Real‐Time PCR System are used in the quantification and qualification of those libraries. For the bioinformatics part, firstly, we filter the low‐quality reads (more than 20% of the qualities of the base are lower than 10), reads with adaptors and reads with unknown bases (N bases more than 5%) to get the clean reads. Then, we map those clean reads onto the reference genome. Finally, we identify DEGs (differentially expressed genes) between samples and do functional annotations (BGI).

### 4.6 Proteomics and bioinformatics analysis

We used iTRAQ (Isobaric tags for relative and absolute quantitation) technology to measure eight samples at one experiment process (protein extraction, enzymolysis, iTRAQ labelling, sample mixing, HPLC separation and LC‐MS/MS analysis) (BGI). After getting raw data, bioinformatics analysis was used in this study. All the proteins with a false discovery rate (FDR) <1% will proceed with downstream analysis including GO and pathway. Furthermore, we also can perform deep analysis based on differentially expressed proteins, including Gene Ontology (GO) and KEGG pathway enrichment analysis.

### 4.7 Association analysis

To investigate the concordance between transcriptome and proteome results in this study, we calculated the Pearson's correlation for these data and created scatter plots. Values were considered significantly positively correlated when R > 0.80, while moderate positive correlation was determined when 0.50 < R < 0.80.

### 4.8 RNA isolation and real‐time PCR

Total mRNAs from rat liver were extracted using TRIzol (Invitrogen) and treated with RNase‐free DNase to remove any residual genomic DNA. Single‐stranded cDNAs were synthesized from 1 μg of total RNA using High‐Capacity cDNA Reverse Transcription Kits (Invitrogen), according to the manufacturer's instructions. The real‐time PCR amplification reactions were conducted in a volume of 20 μl containing 10 μl of SYBR PCR Green Master Mix (Applied Biosystems), and 2 μl of forward and reverse primers each at a final concentration of 10 pmol/μl and 1 μl of cDNA. The primer sequences used in real‐time PCR were as follows: FAS, forward 5′‐CTG CAG ATA TGC TGT GGA TCA‐3′, reverse 5′‐TTT GGT GTT GCT GGT TGG T‐3′; ACC1, forward 5′‐TCT ATT CGG GGT GAC TTT C‐3′, reverse 5′‐CTA TCA GTC TGT CCA GCC C‐3′; ACSL1, forward 5′‐ATG CCA GAG CTG ATT GAC ATT CGG‐3′, reverse 5′‐CAA GGA CTG CTG ATC TTC GGA CAC‐3′; FABP1, forward 5′‐CTG TGG AAA GGA AAC CTC ATT G‐3′, reverse 5′‐GGT GAT GGT GAG TTT GAC TTT C‐3′; Cyp1a1, forward 5′‐GAT GCT GAG GAC CAG AAG ACC GC‐3′, reverse 5′‐CAG GAG GCT GGA CGA GAA TGC‐3′; Alox15, forward 5′‐CGT TGC TCC TCC TCC AGT CTC C‐3′, reverse 5′‐GTT GCA GAA CCA GGC GTC ATC C −3′; Ehhadh, forward 5′‐TCA GTT GGC GTT CTT GGC TTG G‐3′, reverse 5′‐CCG TTC TGA TGC GCT CTG GAT G‐3′; Eci1, forward 5′‐GCC GAG CGT GCC CTT CAA C‐3′, reverse 5′‐GCC ATC ACT GAG CGA GCC TTG‐3′; Hadh, forward 5′‐CAC CGA TGA CCA GCC AGA AGA C‐3′, reverse 5′‐GGC ACC AAG AGA CGG TTC ACG‐3′; CAT, forward 5′‐CGT CAC TCA GGT GCG GAC ATT C −3′, reverse 5′‐TCA GGT GGT TGG CAA TGT TCT CAC‐3′; CPT2, forward 5′‐CAA CAT CCT GTC CAC CAG CAC TC‐3′, reverse 5′‐GCA GCC TAT CCA GTC ATC GTG AAC‐3′; GSTA2, forward 5′‐TGC TGG AAC TTC TCC TCT ATG TTG‐3′, reverse 5′‐AGG CTG CTG ATT CTG CTC TTG‐3′; β‐actin, forward 5′‐CCT GTG GCA TCC ATG AAA CTA C‐3′, reverse 5′‐CCA GGG CAG TAA TCT CCT TCT G‐3′. Thermal cycling and fluorescence detection were performed on an ABI Prism 7500HT Sequence Detection System (Applied Biosystems). Thermal cycling was carried out at 95°C for 10 min, followed by 40 cycles of denaturing at 95°C (15 s), annealing at 60°C (30 s) and extension at 72°C (30 s). Data were analysed using the comparative 2^−ΔΔ^ *^C^* ^t^ method, taking β‐actin as the normalizer.

### 4.9 Protein measurement and Western blotting

The protein expressions of CAT, GSTA2, FAS, ACC1, FABP1, CPT2, ACSL1, FOXO1, NF‐kB and SIRT1 were determined by Western blot analysis. Antibodies against CAT, FAS, ACC1 and FABP1 were purchased from Cell Signaling Technology, antibodies against SIRT1, NF‐kB and β‐actin were purchased from Affinity Biosciences, antibodies against CPT2 and ACSL1 were purchased from Abcam, antibodies against GSTA2 were purchased from Biorbyt, and antibodies against FOXO1 were purchased from Wanleibio. The liver was placed in RIPA lysis buffer (Beyotime Biotechnology, China) and centrifuged (20627 *g* for 15 min) to remove impurities. Protein was quantitated using the Bradford method (Kruger, 1994). Equal amounts of protein were separated by SDS‐PAGE and electrotransferred onto polyvinylidene difluoride (PVDF) membranes. Non‐specific binding was blocked with 5% BSA with 0.05% Tween in triethanolamine‐buffered saline solution (TBS) and incubated with primary antibodies at 4°C overnight, followed by appropriate secondary antibodies for 1 h at 30°C. The membranes were washed three times with TBST (TBS containing 0.05% Tween). The signal was amplified by colour development using the ProtoBlot II AP System with a stabilized substrate (Promega Corporation). For each study, Western blot analysis was conducted three times and representative blots were shown.

### 4.10 Statistical analysis

Statistical analyses were carried out using the Statistical Package of the Social Sciences (SPSS) 23.0 software. Data are presented as mean SD. Two‐factor repeated‐measures ANOVA was used to analyse variables over time and with EGCG. Paired samples t test was used for all comparisons of sample averages. All *p* values were 2‐tailed, and a *p *< 0.05 was considered significant for all statistical analyses in this study.

## CONFLICT OF INTEREST

The authors declare that there is no conflict of interest.

## AUTHOR CONTRIBUTIONS

HY, YL and YN designed and performed the experiments, analysed the data and wrote the manuscript; YG, FL, DZ, QZ and YW performed the experiments and analysed the data; HY, YL and JL analysed the data and wrote the manuscript.

## Supporting information

Supplementary Material

## Data Availability

The data that support the findings of this study are available from the corresponding author upon reasonable request.
