# Supplementary material for: The phytochemical epigallocatechin gallate prolongs the lifespan by improving lipid metabolism, reducing inflammation and oxidative stress in high‐fat diet‐fed obese rats
Source: Aging Cell. 2020 Jul 30;19(9):e13199. doi: 10.1111/acel.13199 (PMC7511879; doi:10.1111/acel.13199)
Supplement: Supplementary file 1 — Supplementary Material [file ACEL-19-e13199-s001.doc]

**Supporting information**

**Table S1** The major pathologies identified at necropsy in rats

| Organ | Pathway | NC (n) | HF (n) | EGCG (n) |
| --- | --- | --- | --- | --- |
| Kidney | Lumpy | 4 | 10 | 6 |
|  | Vesicular | 1 | 3 | 2 |
| Heart | Enlarged | 0 | 1 | 0 |
| Liver | Discolor | 6 | 17 | 7 |
|  | Vesicular | 1 | 2 | 0 |
|  | Tumor | 0 | 1 | 0 |
| Lung | Tumor | 0 | 1 | 0 |
| Subcutaneous | Tumor | 3 | 7 | 2 |
| Aural region | Tumor | 0 | 1 | 0 |
| Intestinal | Tumor | 0 | 1 | 0 |
| Penis | Necrosed/discolor | 2 | 5 | 2 |
| Bladder | Distended | 1 | 3 | 1 |

**Table S2** The major pathologies determined by blinded histopathological analysis in rats

| Tissue | Pathology | NC (n) | HF (n) | EGCG (n) |
| --- | --- | --- | --- | --- |
| Kidney | Fibrosis/Inflammatory cell infiltration | 0 | 3 | 1 |
|  | Chronic pyelonephritis | 4 | 10 | 6 |
|  | Hydropic degeneration | 1 | 3 | 1 |
| Liver | Fatty degeneration | 4 | 14 | 4 |
|  | Hydropic degeneration | 2 | 3 | 2 |
|  | Necrosis | 0 | 2 | 0 |
|  | Congestion | 0 | 2 | 1 |
|  | Inflammatory cell infiltration | 0 | 2 | 0 |
|  | Cancer | 0 | 1 | 0 |
| Lung | Cancer | 0 | 1 | 0 |
| Subcutaneous | Myxoma | 0 | 1 | 0 |
|  | [Granuloma](javascript:;) | 0 | 2 | 0 |
|  | Fibrosis/Inflammatory cell infiltration/Necrosis | 3 | 4 | 2 |

**Table S3** The serum FFA profiles of three groups at 46 and 100 weeks in rats

| Fatty acids | 46 weeks | | | | 100 weeks | | | |  |
| --- | --- | --- | --- | --- | --- | --- | --- | --- | --- |
| （ug/ml） | | NC (n=9) | HF (n=9) | EGCG (n=9) | | NC (n=7) | HF (n=7) | EGCG (n=7) | |
| C14:0 | | 5.48±0.77 | 8.32±0.84^**^ | 6.79±0.85^**##^ | | 2.54±1.12 | 3.45±0.84^*^ | 2.54±1.31^#^ | |
| C16:0 | | 163.50±9.21 | 238.93±16.27^**^ | 163.69±10.89^##^ | | 49.95±7.14 | 77.87±8.54^**^ | 57.96±12.81^##^ | |
| C16:1 | | 53.57±12.05 | 93.29±8.76^**^ | 56.9±7.57^##^ | | 12.51±5.82 | 26.52±5.90^**^ | 20.10±6.30^**#^ | |
| C18:0 | | 268.07±26.75 | 334.79±61.71^**^ | 282.56±49.17^#^ | | 158.60±28.79 | 232.27±57.36^**^ | 185.85±45.94^#^ | |
| C18:1 | | 358.73±55.45 | 447.54±167.82 | 383.68±52.52 | | 188.42±24.89 | 261.78±97.44^*^ | 229.85±71.86 | |
| C18:2 | | 675.94±81.07 | 861.52±158.2^**^ | 650.28±128.67^##^ | | 331.79±92.11 | 425.37±52.31^*^ | 363.75±60.23^#^ | |
| C18:3γ | | 11.25±3.27 | 31.26±3.87^**^ | 8.40±1.10^*##^ | | 2.92±1.29 | 4.79±2.78^*^ | 2.62±1.56^#^ | |
| C18:3α | | 65.47±24.68 | 47.81±15.51^*^ | 65.10±13.43^#^ | | 23.63±12.05 | 17.91±12.45 | 28.98±13.96^#^ | |
| C18:4 | | 0.60±0.29 | 0.42±0.24 | 0.58±0.09^#^ | | 0.31±0.15 | 0.29±0.20 | 0.19±0.14 | |
| C20:2 | | 2.02±0.97 | 1.65±1.23 | 1.85±0.91 | | 2.00±1.09 | 1.53±0.93 | 1.68±1.14 | |
| C20:3 | | 2.26±1.08 | 2.22±0.9 | 1.91±0.62 | | 1.70±1.22 | 1.20±0.74 | 1.12±0.49 | |
| C20:4 | | 151.83±49.39 | 217.24±40.04^**^ | 162.47±24.93^##^ | | 60.62±19.74 | 98.27±32.21^**^ | 76.27±16.21^*#^ | |
| C20:5 | | 1.75±0.49 | 0.88±0.49^**^ | 1.10±0.53^**^ | | 0.68±0.29 | 0.74±0.42 | 0.83±0.42 | |
| C22:4 | | 0.50±0.25 | 0.43±0.25 | 0.51±0.19 | | 0.38±0.26 | 0.55±0.20 | 0.45±0.23 | |
| C22:5 | | 1.62±0.58 | 1.87±0.87 | 0.84±0.14^**##^ | | 0.88±0.39 | 0.63±0.41 | 0.77±0.22 | |
| C22:6 | | 13.03±2.65 | 11.63±3.11 | 12.01±2.56 | | 7.11±1.38 | 5.02±2.21* | 6.16±1.10 | |
| Total SFA | | 437.05±25.49 | 582.04±58.39^**^ | 453.03±47.96^##^ | | 211.09±23.69 | 313.89±59.85^**^ | 246.35±44.48^*##^ | |
| Total MUFA | | 412.31±58.38 | 540.83±163.74^*^ | 440.58±50.93^#^ | | 200.93±23.69 | 288.30±98.58^*^ | 249.95±67.64^*^ | |
| Total PUFA | | 926.27±75.76 | 1176.95±152.03^**^ | 905.04±125.51^##^ | | 432.03±97.29 | 556.31±57.34^**^ | 482.81±50.22^##^ | |
| Total FFA | | 1775.63±88.32 | 2299.81±195.58^**^ | 1798.66±131.99^##^ | | 844.05±93.33 | 1158.50±135.49^**^ | 979.12±107.78^**##^ | |
| Total n-3 FFA | | 80.85±24.08 | 60.74±17.01^*^ | 78.79±13.15^#^ | | 31.73±12.49 | 23.96±11.82 | 36.16±13.64^#^ | |
| Total n-6 FFA | | 845.42±65.65 | 1116.21±150.35^**^ | 826.25±127.59^##^ | | 400.30±102.84 | 532.35±53.01^**^ | 446.65±52.43^##^ | |
| n-6/n-3 | | 10.46 | 18.38 | 10.49 | | 12.62 | 22.22 | 12.35 | |
| LA/ALA | | 10.32 | 18.02 | 9.99 | | 14.04 | 23.75 | 12.55 | |

All values are mean ± SD. ^#^ *P* <0.05, ^##^ *P* <0.01 vs. HF group (ANOVA). ^*^ *P* <0.05, ^**^ *P* <0.01 vs. NC group (ANOVA).

**Figure legends**

**Figure S1** The FFA composition of chow and the effects of EGCG on serum FFA levels. (A) The composition of saturated (SFA), monounsaturated (MUFA), polyunsaturated fatty acids (PUFA) in normal chow and high–fat chow. (B) The ratios of SFA, MUFA, n-6 PUFA, and n-3 PUFA in three groups at 46 weeks and 100 weeks of age (n=9 at 46 weeks, n=7 at 100 weeks).

**Figure S2** KEGG Pathway enrichment analyses for differentially expression genes with in liver transcriptome (n=3)

**Figure S3** Differentially expression proteins detection and functional enrichment analysis on the effects of EGCG in liver proteome. (A) The intersection and aggregate count of differential proteins between two groups. (B) Volcano Plot analysis for the differentially expressed proteins in HF+EGCG vs. NC group. Red dots (upregulated), blue dots (downregulated) and Gray dots (non-regulated). (C) Heap map for classification of the differentially expressed proteins. (D) KEGG pathway enrichment analysis with top 20 enrichment scores in EGCG compared with HF group (n=3). (E) KEGG pathway enrichment analysis with top 20 enrichment scores in HF compared with NC group (n=3).

**Figure S1**


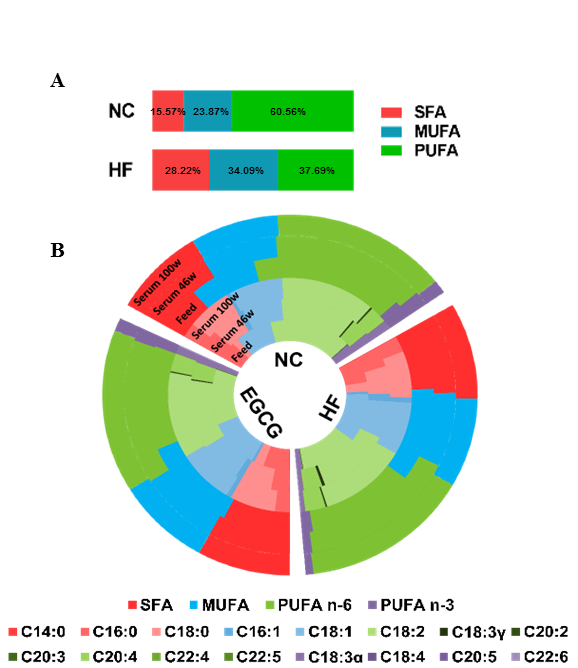


**Figure S2**


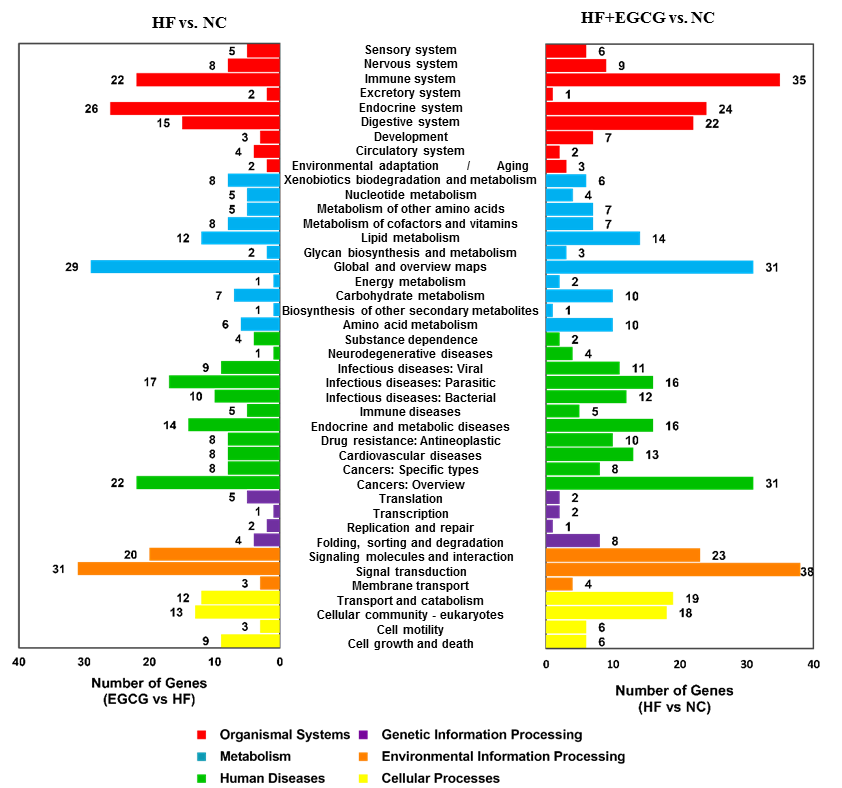


**Figure S3**

**
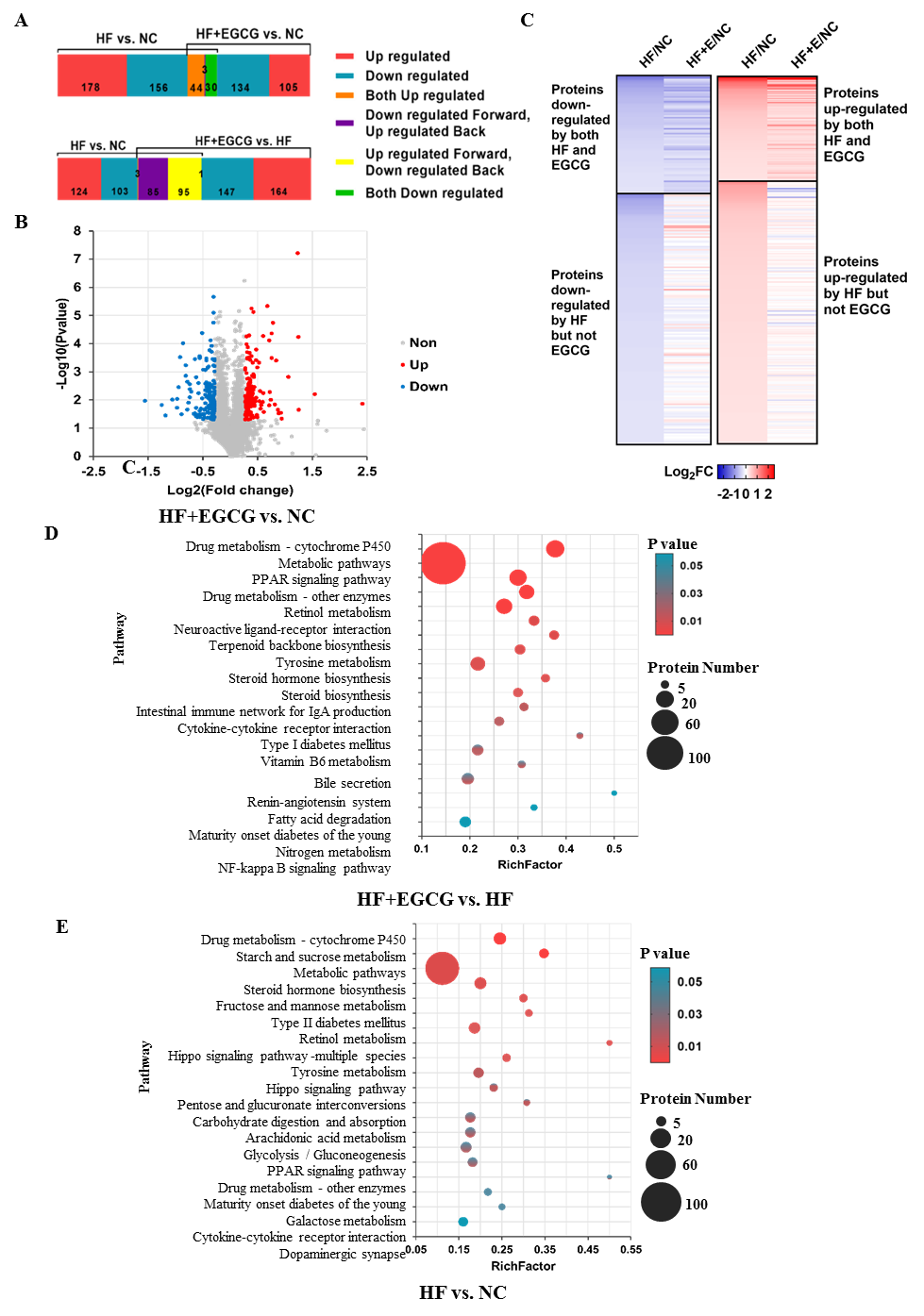
**
